# Modulatory Effects of *Piper sarmentosum* Roxb. Aqueous Extract on Gut Microbiota and Obesity in a High-Fat Diet Rat Model

**DOI:** 10.3390/nu18162667

**Published:** 2026-08-14

**Authors:** Romteera Kittichaiworakul, Patcharawadee Thongkumkoon, Chutima S. Vaddhanaphuti, Worarat Rojanaverawong, Sawannee Sutheeworapong, Rawiwan Wongpoomchai, Sivamoke Dissook

**Affiliations:** 1 Department of Biochemistry, Faculty of Medicine, Chiang Mai University, Chiang Mai 50200, Thailand; 2 Center of Multidisciplinary Technology for Advanced Medicine (CMUTEAM), Faculty of Medicine, Chiang Mai University, Chiang Mai 50200, Thailand; 3 Cluster of Innovative Research for Translational Medicine in Non-Communicable Diseases (CIRTM-NCDs), Faculty of Medicine, Chiang Mai University, Chiang Mai 50200, Thailand; 4 Innovative Research Unit of Epithelial Transport and Regulation, Department of Physiology, Faculty of Medicine, Chiang Mai University, Chiang Mai 50200, Thailand; 5 Office of Research Administration, Chiang Mai University, Chiang Mai 50200, Thailand; 6 Systems Biology and Bioinformatics Research Unit, Pilot Plant Development and Training Institute, King Mongkut’s University of Technology Thonburi, Bangkok 10150, Thailand

**Keywords:** obesity, high-fat diet, *Piper sarmentosum* Roxb., simvastatin, gut microbiome, HPLC phenolic profile, combination therapy

## Abstract

**Background/Objectives:** Obesity and its metabolic comorbidities, including dyslipidemia and non-alcoholic fatty liver disease (NAFLD), are a growing global health burden, and current pharmacotherapies have limited efficacy and notable adverse effects. The gut microbiome has emerged as a key regulator of host metabolism, and its dysregulation is implicated in obesity. This study investigated the therapeutic potential of *Piper sarmentosum* Roxb. aqueous extract (PSRW) on metabolic dysfunction and gut microbiota composition in a high-fat diet (HFD)-induced obese rat model. **Methods:** HFD-induced obese male Wistar rats received PSRW, simvastatin, or their combination for 12 weeks. Serum lipids (TC, TG, HDL, LDL), liver enzymes (AST, ALT), and renal markers were measured, and gut microbiota were profiled by 16S rRNA (V4) sequencing with PICRUSt2/KEGG functional prediction. The phenolic composition of the same extract was characterized by HPLC in a companion study. **Results:** PSRW did not cause weight loss but significantly lowered serum TC, AST, and ALT, with efficacy comparable to simvastatin and significantly lower TG than control, though not significantly different from HFD. Co-administration of PSRW and simvastatin produced a greater reduction in TC and liver enzymes than either agent alone. HFD shifted microbial community structure and enriched pro-inflammatory phyla such as *Desulfobacterota*; PSRW partially reversed these changes, suppressing obesogenic *Lachnospiraceae* genera while enriching beneficial microbes including *Dwaynesavagella*, and functionally reprogrammed taxa such as *Kineothrix* and *Muribaculum* from detrimental to protective associations. HFD enriched microbial glycosphingolipid biosynthesis, whereas the PSRW simvastatin combination uniquely enhanced glycine, serine, and threonine metabolism. **Conclusions:** *P. sarmentosum* extract ameliorates HFD-induced dyslipidemia and hepatotoxicity, and these effects are accompanied by remodeling of the composition and predicted functional capacity of the gut microbiome. Its ability to act in combination with statins highlights its potential as a candidate complementary therapy for hyperlipidemia and NAFLD.

## 1. Introduction

Obesity is a global health concern and a chronic metabolic disorder mainly driven by an imbalance between energy intake and energy expenditure, resulting in the excessive accumulation of adipose tissue in the body [1]. Pathologically, obesity involves expansion of white adipose tissue by adipocyte hypertrophy and hyperplasia, along with ectopic fat deposition in organs such as the liver, skeletal muscle, kidneys, and heart [2,3]. The pathogenesis of obesity involves a complex interplay of genetic predispositions and non-genetic elements, including environmental, behavioral patterns, and developmental factors [4,5]. These factors heighten the risk of developing numerous obesity-associated comorbidities, such as hypertension, stroke, dyslipidemia, insulin resistance, type 2 diabetes mellitus, myocardial infarction, and various cancers [6,7]. Visceral adiposity has also been linked to a wider spectrum of chronic complications, including cognitive decline and Alzheimer’s disease, increasing the clinical urgency for effective management [8]. Conventional pharmacotherapies for obesity-associated dyslipidemia and NAFLD, particularly statins such as simvastatin and rosuvastatin, are effective but limited by dose-dependent adverse effects, statin intolerance, drug–drug interactions, and an inability to remodel the gut microbiome [9,10]. These limitations motivate complementary phytotherapeutic strategies that act through host- and microbe-targeted mechanisms distinct from HMG-CoA reductase inhibition.

Recent research on the gut microbiota (GM) has emerged as a key environmental factor contributing to the onset and progression of obesity [11,12]. The GM refers to the diverse and dynamic community of microorganisms, predominantly bacteria, that colonize the gastrointestinal (GI) tract [13,14]. This complex microbial ecosystem is integral to host physiology, playing critical roles in regulating host metabolism, maintaining energy homeostasis, modulating immune function, and facilitating nutrient absorption [15,16,17]. Imbalance in GM composition and function, known as gut dysbiosis, can be triggered by dietary patterns and lifestyle factors and has been consistently associated with the pathophysiology of metabolic disorders, including obesity [18,19].

One mechanism by which dysbiosis contributes to obesity involves the disruption of intestinal barrier integrity, leading to increased gut permeability [20]. This “leaky gut” condition permits the translocation of endotoxins, such as lipopolysaccharides, from the gut lumen into the systemic circulation, a phenomenon termed metabolic endotoxemia [21,22]. These gut-derived components trigger chronic low-grade inflammation, a hallmark of obesity and its related metabolic diseases [22,23]. In most recent research, GM influences host immune modulation, which is increasingly recognized as a pathway linking microbial composition to obesity pathophysiology. Obesity has also been associated with distinct shifts in microbial profiles, most notably a reduced overall bacterial diversity and an altered ratio of the two dominant phyla, Firmicutes and Bacteroidetes [24,25]. Several bacterial genera with beneficial metabolic functions, such as *Akkermansia*, *Faecalibacterium*, *Oscillibacter*, *Alistipes*, and *Lactobacillus reuteri*, are depleted in individuals with obesity. In contrast, a higher relative abundance of beneficial microbes, including species of *Bifidobacterium* and *Lactobacillus*, alongside the archaeon *Methanobrevibacter smithii*, has been reported more frequently in healthy, lean individuals [26,27].

In light of these findings, attention has increasingly turned toward traditional medicine and natural products as potential modulators of GM composition and metabolic health [28,29]. *Piper sarmentosum* Roxb. (hereafter abbreviated *P. sarmentosum*; aqueous extract designated PSRW; locally known as Cha Phul), an edible medicinal plant in the family *Piperaceae*, is traditionally used in various Asian countries for treating gastrointestinal disorders, gastritis, hyperglycemia, and diabetes mellitus. The therapeutic potential of *P. sarmentosum* is attributed to its rich profile of bioactive compounds, which includes phenylpropanoids, flavonoids, amides, alkaloids, and lignans. *P. sarmentosum* has shown inhibitory effects on adipogenesis by reducing fat mass and intracellular triglyceride accumulation in preadipocytes. It also exhibits potent hypoglycemic activity in diabetic-induced rats by reducing plasma glucose levels, along with antioxidant, anti-inflammatory, anti-obesity, and antibacterial activity [30,31,32]. A recent companion study by our group analyzed the same aqueous extract (PSRW) by HPLC and identified its major compounds, including quercetin/trans-cinnamic acid, epicatechin, chlorogenic acid, caffeine, and gallic acid. That study also showed potent lipid-lowering activity in vitro and in vivo, reducing cholesterol levels in serum, intestinal, and hepatic tissues to a degree comparable to ezetimibe [33].

Despite these promising findings, the effects of *P. sarmentosum* on the gut microbiota remain poorly understood. Building on this evidence, the present study aimed to (i) evaluate the in vivo metabolic effects of *P. sarmentosum* Roxb. aqueous extract in a high-fat diet-induced obese male Wistar rat model on body weight, serum lipid profile (TC, TG, HDL, LDL), liver enzymes (AST, ALT), and renal markers (BUN, creatinine); (ii) profile gut microbiota composition (16S rRNA, V4 region); (iii) benchmark PSRW against the standard lipid-lowering drug simvastatin and test whether their co-administration confers additive or synergistic benefit; and (iv) infer microbial functional pathways using PICRUSt2/KEGG to identify candidate mechanisms underlying the host–microbe metabolic effects.

## 2. Materials and Methods

### 2.1. Plant Preparation and Extraction

Fresh leaves of *Piper sarmentosum* Roxb. were collected from Terd Walai Farm, located in Nong Tong Subdistrict, Hang Dong District, Chiang Mai Province, Thailand (approximate GPS coordinates: 18.6856° N, 98.8853° E). The plant was identified as a voucher specimen (No. 0023333) deposited at the Herbarium, Faculty of Pharmacy, Chiang Mai University, and authenticated by the curator of the Herbarium in accordance with standard taxonomic procedures. The leaves were thoroughly washed and blended, then extracted with hot distilled water (1:5 *w*/*v* leaves-to-water ratio, boiled at 95–100 °C) for 15 min. The filtrate was centrifuged (9000 rpm, 10 min) to remove insoluble debris. The aqueous extract was subsequently lyophilized and stored at −20 °C before analysis. The mean extraction yield was 4.8 ± 0.3% (*w*/*w*, lyophilized extract per fresh leaf mass), consistent with our companion HPLC-characterized batch [33].

### 2.2. Phytochemical Characterization of PSRW by HPLC

The PSRW used here was prepared from the same batch and protocol as our companion study [33], in which its phenolic composition was fully characterized by HPLC [33]. Briefly, the major phenolic constituents were quercetin/trans-cinnamic acid (14.51 mg/g), epicatechin (5.80 mg/g), chlorogenic acid (2.75 mg/g), caffeine (1.13 mg/g), and gallic acid (0.44 mg/g), giving a total phenolic content of 24.63 mg/g; full instrumental conditions are reported in [33]. This phenolic fingerprint provides the chemical basis for the antioxidant, anti-inflammatory, and lipid-lowering activities discussed below. Phenolic compounds account for 24.63 mg/g of the administered aqueous fraction; the remaining mass was not characterized in the present work and is expected to comprise mainly soluble carbohydrates, organic acids, free amino acids and peptides, and minerals. The contribution of these non-phenolic constituents to the effects reported here therefore cannot be excluded, and compositional profiling of this fraction is a priority for follow-on work.

### 2.3. Animals

Male Wistar rats (4–5 weeks old, conventional health status) were purchased from Nomura Siam International (Bangkok, Thailand). To limit external microbial variation, all rats were obtained from a single vendor and a single shipment and were housed in one room under identical conditions. Rats were assigned to six experimental groups, with *n* = 5 rats per group analyzed (two cages of two, one cage of one). Sample size was determined by a priori power analysis (G*Power version 3.1; effect size f = 0.6, α = 0.05, power = 0.80) based on lipid and liver-enzyme endpoints reported in prior HFD studies of *Piper*-related extracts, consistent with the 3Rs principle (Replacement, Reduction, Refinement) endorsed by our institutional ethics committee. Male animals were selected to minimize the confounding influence of cyclical estrogen fluctuations, which independently modulate lipid metabolism, hepatic steatosis, and gut microbiota in female rodents; the implications of this restriction, including the inability to generalize to female biology, are acknowledged in the Limitations section.

All rats were group-housed and acclimatized for one week under standard laboratory conditions prior to the start of the experiment. Animals were housed in a controlled environment with a 12 h light/dark cycle and maintained at a constant temperature of 25 ± 1 °C, with ad libitum access to standard chow and water throughout acclimatization.

### 2.4. Diet

Rats were maintained on standard chow ad libitum during the one-week acclimatization period. Following acclimatization, the high-fat diet (HFD) was custom-formulated to deliver approximately 60% of total energy from fat (mainly lard and soybean oil), 20% from carbohydrate, and 20% from protein, providing ≈5.24 kcal/g, in line with published HFD-induced obesity models [34].

### 2.5. Induction of Obesity in Rats

After acclimatization, rats were allocated to a normal-diet (Control) group or a high-fat-diet (HFD) group by stratified randomization based on baseline body weight, and maintained on their respective diets for 12 weeks. Cage positions on the rack were not rotated during the study. According to Rojanaverawong et al. [33], obesity was operationally defined by two criteria, including a body weight at least 20% greater than that of age-matched chow-fed controls after 12 weeks of HFD feeding, and elevated serum total cholesterol (TC) and/or LDL relative to controls, confirmed at the end of the 12-week induction period prior to randomization for treatment. Animals were recorded every other day for body weight, food intake, and clinical signs of distress. No adverse events requiring early euthanasia or removal from the study were observed. No blinding was performed during the conduct of the experiment or data analysis.

**Figure 1:**
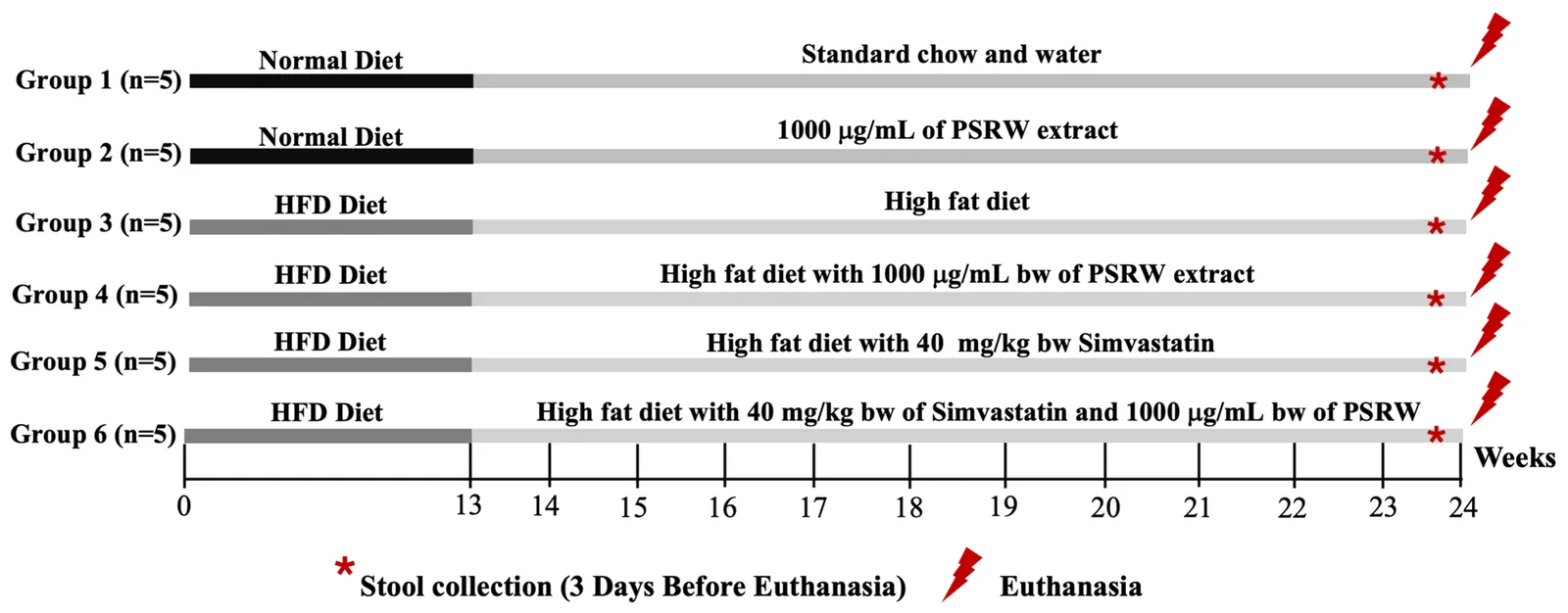
Study-design schematic for the high-fat diet-induced obesity model, including the timeline for administering PSRW and HFD-based treatments to each group.

In week 13, all rats were further subdivided into six groups (*n* = 5 rats per group) and received daily oral gavage as shown in Figure 1. PSRW was suspended in sterile distilled water immediately before administration and delivered by oral gavage as a 1000 µg/kg body weight (bw)/day dose of PSRW extract; simvastatin was prepared in distilled water and delivered by oral gavage at 40 mg/kg bw/day. The PSRW concentration was selected based on a 1000 µg/kg bw radiolabelled in vivo dose previously shown to be effective in our companion study [33], and prior reports of efficacy of *P. sarmentosum* extracts in rats [33]. The selected dose of 40 mg/kg simvastatin was consistent with effective lipid-lowering doses reported in our prior HFD-induced hepatic steatosis in rats [35] and anti-inflammatory effects in hypercholesterolemia patients [36]. Moreover, a half-dose of 40 mg/kg simvastatin also altered gut microbiota in 8-week HFD-induced rats [37]. A single, mechanistically justified dose, rather than a multi-dose response, was used in this first-in-rat microbiome study to maintain power per group, minimize animal use under the 3Rs framework, and concentrate analytical capacity on deep 16S rRNA sequencing. Full dose–response characterization is planned for a follow-on study and is listed as a limitation.

All groups received continuous treatment for 12 weeks. Three days before the end of the study, stool samples were collected from all rats for microbiome analysis. Fresh fecal pellets were collected directly from each rat by attaching a sterile microcentrifuge tube to the animal’s anus, preserved in DNA/RNA Shield solution, and stored at −80 °C to maintain microbial nucleic-acid integrity. At the end of the study, rats were euthanized by intraperitoneal injection of thiopental sodium (100 mg/kg body weight). Blood samples were collected and centrifuged at 3000× *g* for 10 min to separate serum, which was stored at −20 °C until further analysis.

### 2.6. Chemicals and Reagents

Commercial colorimetric assay kits for glucose, triglycerides (TG), total cholesterol (TC), low-density lipoprotein (LDL), and high-density lipoprotein (HDL), as well as enzymatic assay kits for aspartate aminotransferase (AST), alanine aminotransferase (ALT), blood urea nitrogen (BUN), and creatinine were obtained from Erba Lachema (Brno, Czech Republic). Simvastatin was acquired from Greater Pharma Manufacturing Co., Ltd., Nakhon Pathom, Thailand. DNA/RNA Shield reagent was purchased from Zymo Research Corp (Irvine, CA, USA). The TIANamp Stool DNA Kit (Cat. No. 4992205) was supplied by Tiangen Biotech (Beijing, China). The MetaVX 16S rRNA Library Preparation Kit and Illumina-compatible sequencing reagents were obtained from GENEWIZ Inc. (South Plainfield, NJ, USA). Simvastatin (analytical grade), HPLC-grade acetonitrile, acetic acid, and all nineteen phenolic acid/flavonoid standards used for HPLC quantification of PSRW were purchased from Sigma-Aldrich (St. Louis, MO, USA). All other chemicals and solvents were of analytical or HPLC grade.

### 2.7. Determination of Metabolic Blood Parameters

Quantitative measurements of glucose, triglycerides (TG), total cholesterol (TC), LDL, and HDL were performed using commercial colorimetric assay kits (Erba Lachema, Brno, Czech Republic) at a wavelength of 500 nm. Aspartate aminotransferase (AST), alanine aminotransferase (ALT), blood urea nitrogen (BUN), and creatinine were quantified using commercial enzymatic assay kits from Erba Lachema (Brno, Czech Republic).

### 2.8. Bacterial Genomic DNA Extraction

All stool samples were provided in DNA/RNA Shield reagent (Zymo Research Corp., Irvine, CA, USA) and stored at −80 °C until further analysis. Total genomic DNA was extracted from approximately 180–200 mg of wet stool sample using the TIANamp Stool DNA Kit (Cat. No. 4992205, Tiangen, Beijing, China), according to the manufacturer’s instructions. DNA concentration and purity were quantified using a NanoDrop spectrophotometer (Thermo Fisher Scientific, Waltham, MA, USA). The OD260/OD280 ratio of all samples was 1.80–2.00, indicating acceptable DNA purity for downstream molecular analysis.

### 2.9. Library Preparation and Sequencing

The sequencing library was constructed using a MetaVX Library Preparation Kit (GENEWIZ, Inc., South Plainfield, NJ, USA). For each sample, 20–30 ng of total DNA was used to amplify the V4 hypervariable region of the bacterial 16S rRNA gene. The forward primer was 515F (GTGYCAGCMGCCGCGGTAA), and the reverse primer was 806R (GGACTACNVGGGTWTCTAAT). PCR amplification was carried out using the following thermal cycling conditions: 3 min denaturation at 94 °C; 26 cycles of 5 s at 95 °C, 90 s annealing at 57 °C, 10 s elongation at 72 °C, and a final extension at 72 °C for 5 min. Indexed adapters were added to the amplicons through limited-cycle PCR. The final libraries were sequenced on a NextSeq 2000 platform (Illumina, San Diego, CA, USA) at GENEWIZ, Inc. (South Plainfield, NJ, USA). Automated cluster generation and paired-end sequencing (250/300 bp) with dual reads were performed according to the manufacturer’s protocols.

### 2.10. Microbiome Data Analysis

Raw Illumina microbial data were imported and processed using the Quantitative Insights Into Microbial Ecology 2 (QIIME2) software, version 2022.2. Quality control and denoising were performed using the q2-dada2 plugin to remove low-quality reads, including short or long sequences and those containing primer mismatches, uncorrectable barcodes, or ambiguous bases. Amplicon sequence variants (ASVs) were then inferred and compiled into a feature table. Taxonomic classification of ASVs was conducted using the QIIME2 feature-classifier plugin, implemented with a Naïve Bayes machine-learning classifier pre-trained on the Greengenes2 database (release 2022.10; 99% similarity), targeting ASVs from the 515F/806R region of the 16S rRNA gene. To normalize sequencing depth across samples, rarefaction was applied to equalize the number of reads. Taxonomic abundances were then assigned using the classify-sklearn plugin.

### 2.11. Diversity Analysis

Taxonomic abundance data at genus and species levels were used to calculate microbial diversity metrics. Alpha diversity indices included Shannon’s diversity and Chao1 richness. Beta diversity was assessed using Bray–Curtis dissimilarity, the Jaccard index, and unweighted and weighted UniFrac phylogenetic distances. Principal coordinates analysis (PCoA) was used to visualize clustering of samples according to their microbiome profiles. All analyses were performed using the *vegan* (v2.6-10) and *phyloseq* (v1.50.0) packages in R v4.4.3. The *ggplot2* (v4.0.3) package was used to generate microbial diversity plots, stacked bar graphs, and box plots of relative abundance.

### 2.12. Enrichment Analysis of Microbial Metabolic Function

Microbial functional prediction was performed using Phylogenetic Investigation of Communities by Reconstruction of Unobserved States (PICRUSt2, v2.3.0b0), which estimates microbial metabolic functions, including KEGG pathways, based on 16S rRNA gene sequences. The predicted functional profiles were normalized using estimated 16S rRNA gene copy numbers. Differential abundance analysis of functional pathways between experimental groups was conducted using Statistical Analysis of Taxonomic and Functional Profiles (STAMP, v2.1.3). Welch’s two-sided t-test was applied, and pathways with a *p*-value < 0.05 were considered significantly different. The mean proportion value was used to indicate the relative enrichment of specific functional pathways within each group.

### 2.13. Statistical Analysis

Data are presented as means ± standard deviation (SD). Statistical analyses and data visualization were performed using R v4.4.3. Given the modest group size (*n* = 5) and the non-Gaussian distribution observed in several microbial relative-abundance variables (assessed by the Shapiro–Wilk test), non-parametric tests were chosen in preference to ANOVA-based parametric tests to avoid violating normality assumptions. Alpha diversity was compared between groups using the Kruskal–Wallis test, with a significance threshold of *p* < 0.05. Beta diversity differences were assessed using permutational multivariate analysis of variance (PERMANOVA), also with a significance threshold of *p* < 0.05, as PERMANOVA is robust to multivariate non-normality and dispersion in ecological community data. For other group comparisons, the Wilcoxon rank-sum test was applied at a 95% confidence level to evaluate both within- and between-group differences. Boxplots and PCoA plots were generated using the *ggplot2* (v4.0.3) package, and *p*-values were annotated using the *ggpubr* (v0.6.2) package. For lipid and liver-enzyme variables in Table 1, between-group differences were further evaluated by one-way ANOVA with Bonferroni post hoc correction following confirmation of approximate normality.

## 3. Results

### 3.1. Effect of PSRW on Metabolic Biomarkers in High-Fat Diet-Induced Obese Rats

As shown in Table 1, rats in the HFD group showed significantly higher final body weight compared with the control group. Furthermore, TC and LDL, as well as AST and ALT levels, were markedly elevated in the HFD group relative to controls. These findings confirm the induction of obesity-associated metabolic disturbances in this model. Administration of *P. sarmentosum* Roxb. (PSRW) extract did not lead to a statistically significant reduction in body weight. However, PSRW treatment resulted in significant decreases in TC, AST, and ALT levels compared with the HFD group. Notably, TG did not differ significantly between the HFD and control groups. TG in the HFD + PSRW group was significantly lower than the control but not significantly different from the HFD group. Treatment with simvastatin alone, as well as in combination with PSRW, also significantly decreased TC, AST, and ALT levels compared with the HFD group.

### 3.2. Effect of PSRW on Gut Microbiota Composition at the Taxonomic Level

**Figure 2:**
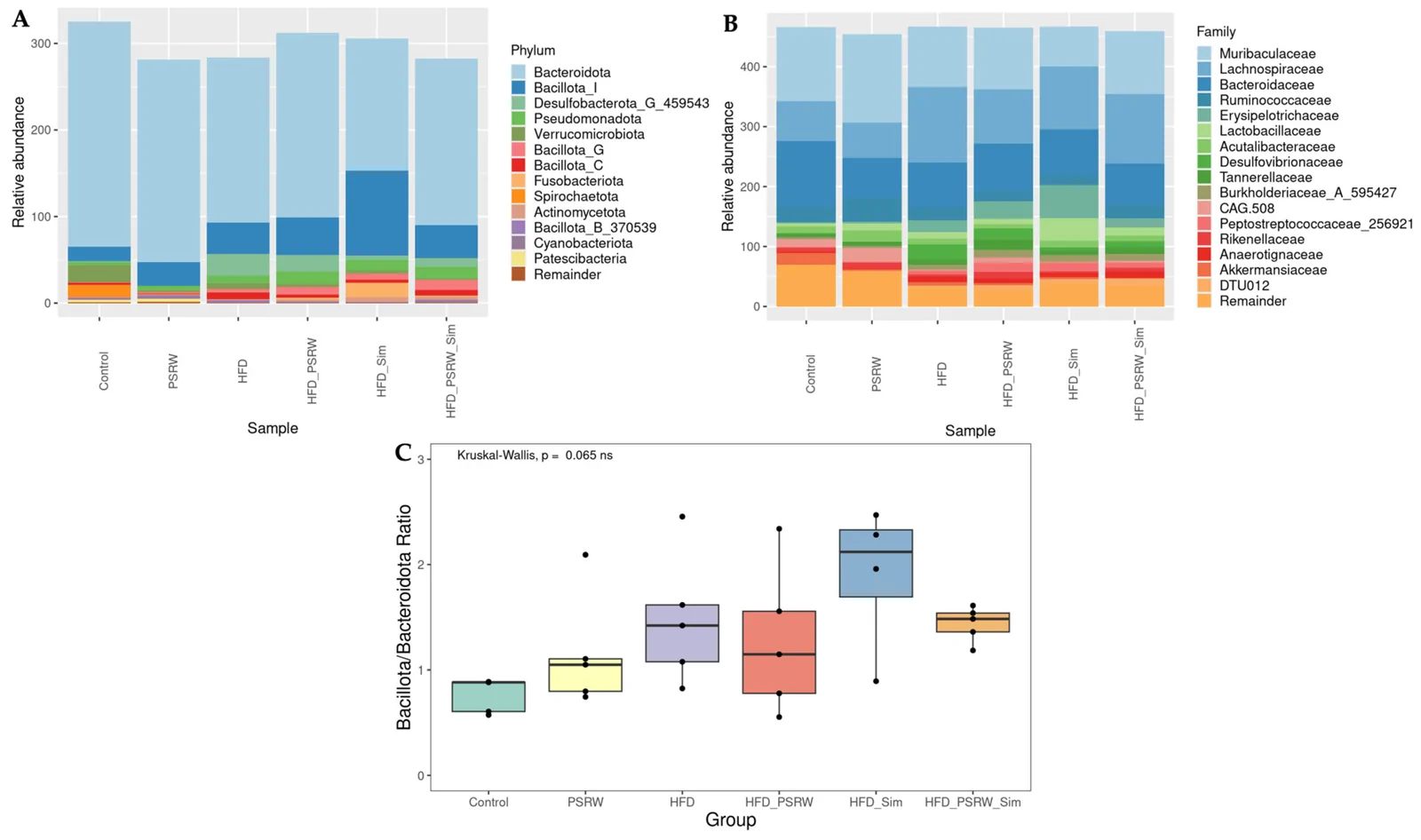
Relative abundance of bacteria across conditions; each group included *n* = 5 rats. (**A**) Relative abundance of microbial compositions at the phylum level. (**B**) Relative abundance of microbial compositions at the family level. (**C**) *Bacillota*-to-*Bacteroidota* ratio across groups.

Microbial structure across all samples was represented by relative-abundance profiles at different taxonomic levels. At the phylum level, *Bacillota* and *Bacteroidota* (formerly *Firmicutes* and *Bacteroidetes*) predominated, accounting for 50.12% and 41.45% of the average microbial composition, respectively (Figure 2A). At the family level, the five taxa with the highest average relative abundance across all groups were *Muribaculaceae* (21.58%), *Lachnospiraceae* (18.73%), *Bacteroidaceae* (15.82%), *Oscillospiraceae_88309* (7.35%), and *Ruminococcaceae* (4.82%) (Figure 2B). However, the *Bacillota*-to-*Bacteroidota* ratio did not differ significantly between groups (Figure 2C), suggesting a relatively stable distribution of these major phyla. Moreover, the HFD group demonstrated an increased relative abundance of *Bacillota_A_368345*, *Desulfobacterota_G_459543*, and *Pseudomonadota*, while the abundance of *Bacteroidota* and *Verrucomicrobiota* was reduced compared with the control group.

### 3.3. Effect of PSRW on Microbial Diversity and Composition

**Figure 3:**
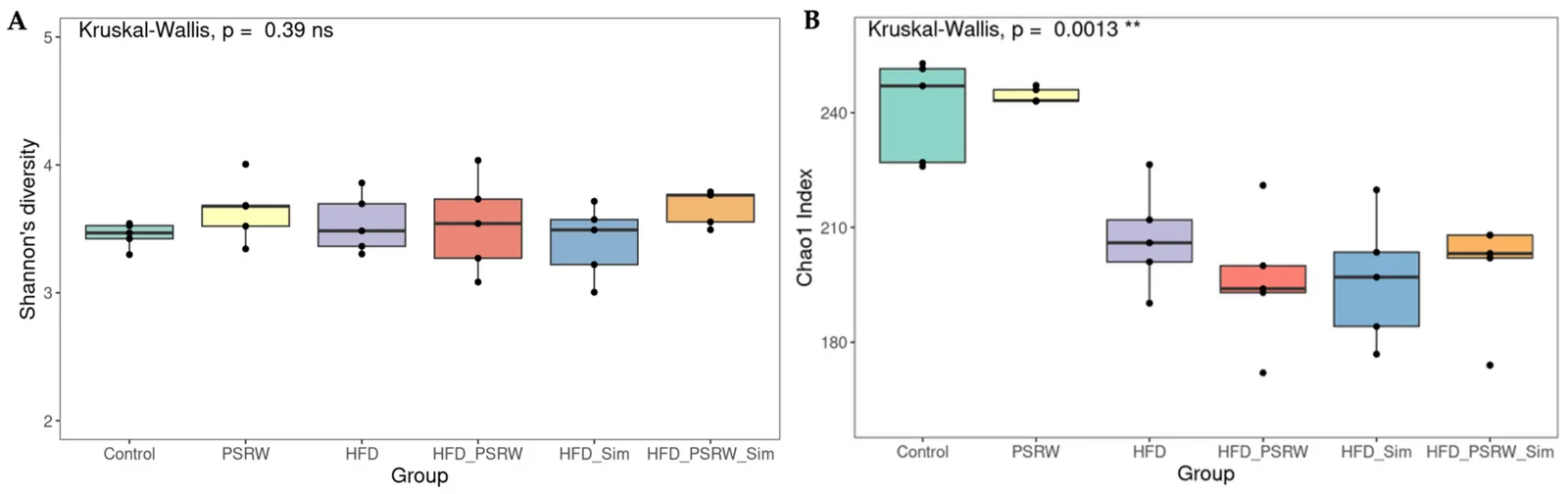
Alpha diversity within control, PSRW, HFD, and HFD-based treatment groups. (**A**) Shannon diversity index (*p* = 0.39). (**B**) Chao1 richness index (*p* = 0.0013). Each group included *n* = 5 rats; statistical significance was determined using the Kruskal–Wallis test. ns, not significant; ** *p* < 0.01.

Alpha diversity analyses, reflecting the microbial community within each group, are shown in Figure 3A,B. Microbial diversity was assessed using the Shannon and Chao1 indices to quantify microbial evenness and richness, respectively. As shown in Figure 3A, no statistically significant differences in Shannon diversity were observed among groups (Kruskal–Wallis, *p* = 0.39), indicating that microbial evenness was not notably altered by the HFD or treatment interventions.

Conversely, the Chao1 richness index (Figure 3B), which estimates the total number of genera present in each group, revealed significant differences between groups (Kruskal–Wallis, *p* = 0.0013). Microbial richness was significantly reduced in the HFD group compared with the control, indicating that the high-fat diet lowered gut microbial diversity. PSRW-treated groups partially restored richness, although the HFD + PSRW, HFD + Sim, and HFD + PSRW + Sim groups all remained lower than the control. No significant differences were observed among these HFD-based treatment groups.

**Figure 4:**
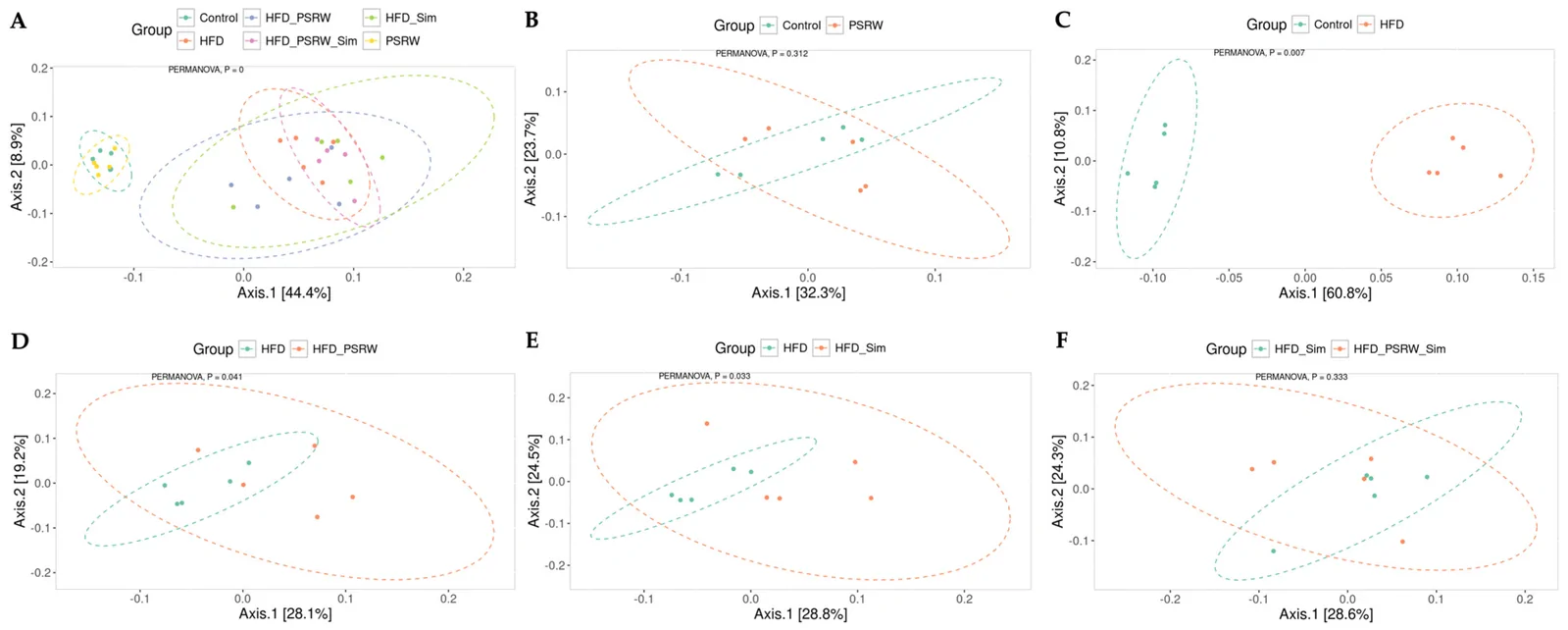
Comparison of bacterial community dissimilarity among groups using principal coordinate analysis (PCoA) based on UniFrac phylogenetic distance. (**A**) PCoA of all groups. (**B**) Control vs. PSRW. (**C**) Control vs. HFD. (**D**) HFD vs. HFD + PSRW. (**E**) HFD vs. HFD + Sim. (**F**) HFD + Sim vs. HFD + PSRW + Sim.

UniFrac phylogenetic distance was used to evaluate beta diversity, indicating compositional differences in microbial communities among groups. The results demonstrated a statistically significant difference in microbial composition among the groups (*p* = 0.000, PERMANOVA), as shown in Figure 4A. Notably, the control and PSRW-treated groups exhibited closely clustered microbial communities (Figure 4B). In contrast, the HFD group showed a distinct shift and greater dispersion, indicating a substantial alteration in gut microbiota compared with the control (Figure 4C). Supplementation with PSRW alone led to significant changes in microbial composition (Figure 4D). Similarly, the Sim group revealed significant differences in microbial composition relative to the HFD group (Figure 4E). However, no significant differences were observed in the HFD + PSRW + Sim group compared with HFD + Sim, indicating that co-treatment with PSRW and Sim did not further alter microbial composition (Figure 4F).

**Figure 5:**
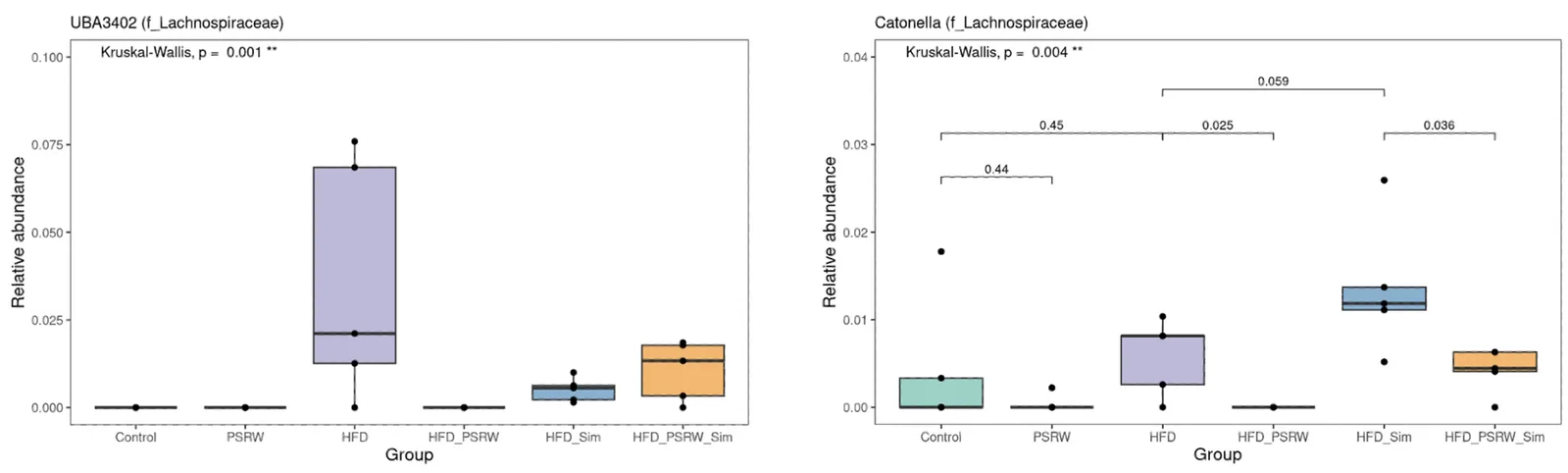

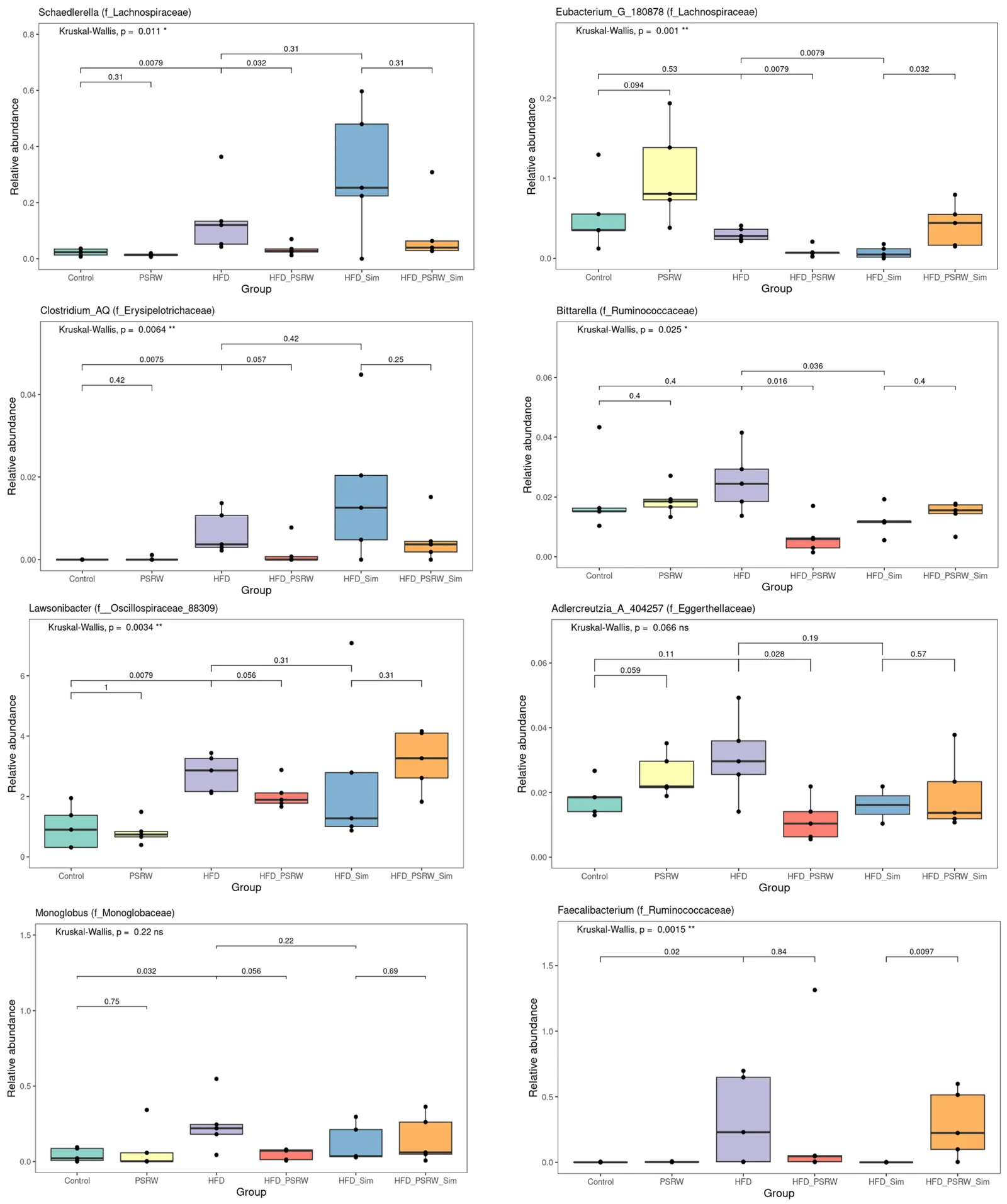

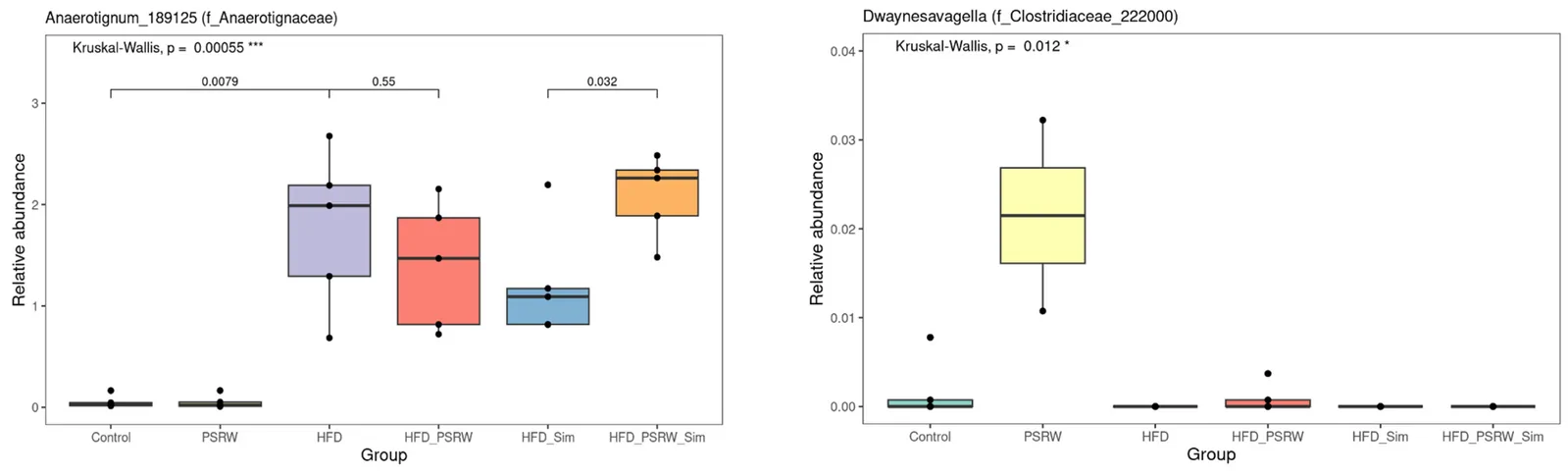
Relative abundance of selected bacterial genera across groups. Boxplots show genus-level relative abundance with Kruskal–Wallis *p*-values; each group included *n* = 5 rats. * *p* < 0.05; ** *p* < 0.01; *** *p* < 0.001; ns, not significant.

While investigating the relative abundance of microbial communities, specific taxa were analyzed across the control, PSRW, HFD, and various HFD-based treatment groups (Figure 5). Compared with the control group, the HFD group exhibited enrichment of several taxa, including *UBA3402*, *Catonella*, *Schaedlerella*, *Clostridium_AQ*, *Bittarella*, *Lawsonibacter*, *Adlercreutzia_A_404257*, and *Monoglobus*. PSRW treatment markedly reduced the relative abundance of these taxa. Furthermore, key microbial taxa within the *Ruminococcaceae* family, such as *Bittarella* and *Faecalibacterium*, were significantly reduced in the HFD-with-PSRW group. Notably, PSRW supplementation was associated with a significant increase in the genus *Dwaynesavagella*. Members of the *Lachnospiraceae* family were consistently the most abundant across all groups. The simvastatin group, which manages dyslipidemia by inhibiting endogenous hepatic cholesterol synthesis, showed reduced levels of *Eubacterium G 180878*, *Bittarella*, *Anaerotignum 189125*, and *Faecalibacterium* compared with the control group. *Catonella*, *Schaedlerella*, *Eubacterium G 180878*, *Anaerotignum 189125*, and *Faecalibacterium* were also decreased in the HFD group co-treated with PSRW and simvastatin. The selected genera are shown in Figure 5.

### 3.4. Correlation of Gut Microbiota and Metabolic Biomarkers

**Figure 6:**
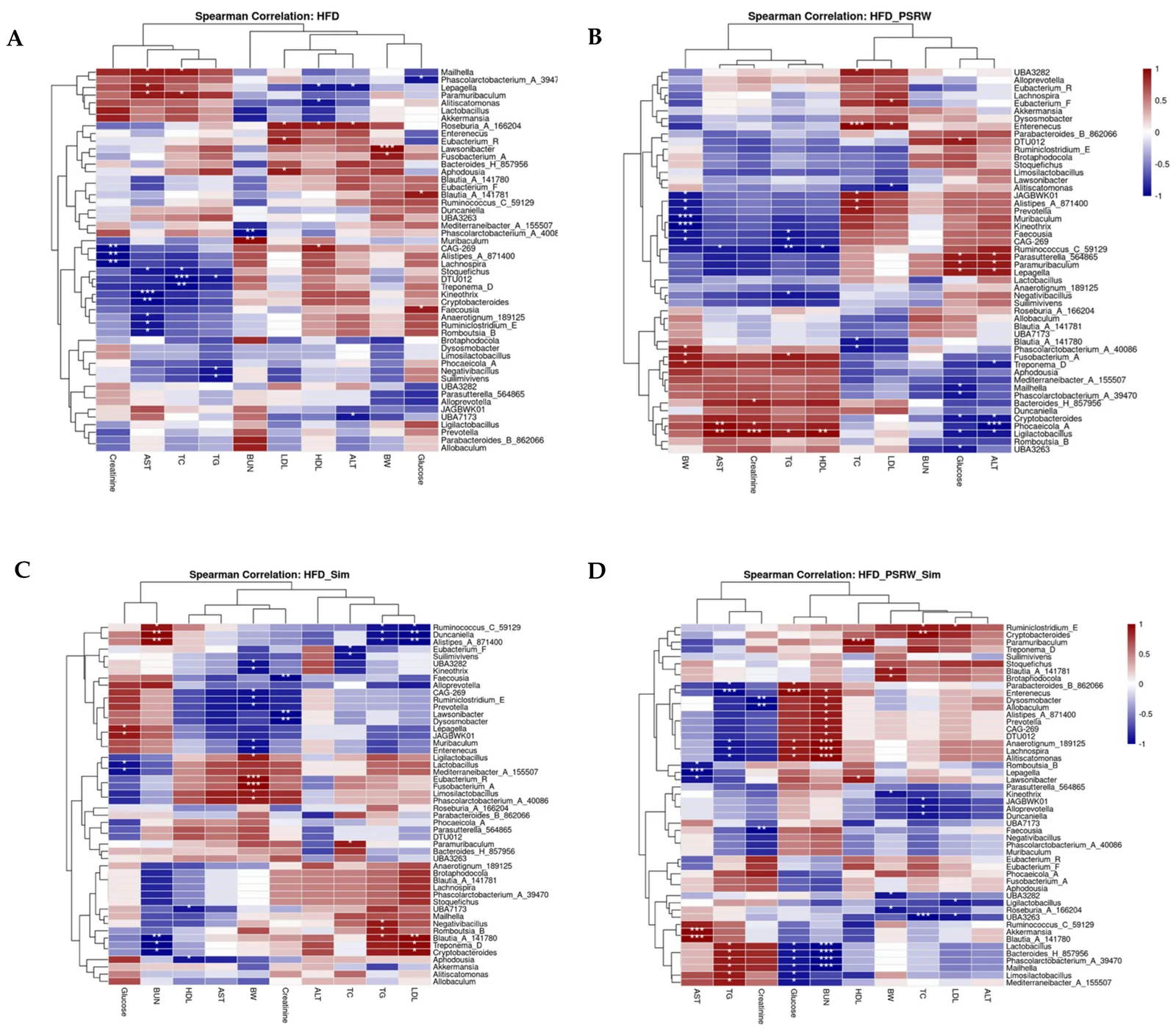
Correlation heatmaps and relative abundance of gut microbiota across the groups. (**A**) HFD. (**B**) HFD + PSRW. (**C**) HFD + Sim. (**D**) HFD + PSRW + Sim. * *p* < 0.05; ** *p* < 0.01; *** *p* < 0.001.

To investigate the relationship between microbial composition and metabolic health, Spearman correlation heatmaps were generated between the relative abundances of microbial genera and key metabolic biomarkers. Results for the HFD control group and the three intervention groups are detailed below. In the HFD group (Figure 6A), the genera *Mediterraneibacter* and *Muribaculum* were significantly positively correlated with TG and TC levels. Similarly, *Fusobacterium* and *Lawsonibacter* showed a significant positive correlation with glucose. In contrast, a large cluster of bacteria, including *Treponema_D*, *Faecousia*, *Kineothrix*, *Cryptobacteroides*, and *Ruminiclostridium_E*, was strongly and significantly negatively correlated with creatinine and AST levels.

The intervention with PSRW resulted in a notable shift in microbial–metabolic correlations. A beneficial association was observed, in which *Kineothrix* and *Muribaculum* were significantly negatively correlated with AST and body weight. However, a prominent cluster including *Ruminococcus C 59129*, *Blautia A 141780*, and *Phascolarctobacterium A 40086* was strongly and significantly positively correlated with BUN and LDL. Additionally, *Phocaeicola A* exhibited a strong positive correlation with ALT levels (Figure 6B). The simvastatin intervention also markedly altered the metabolic relationships of the microbiome: a significant negative correlation was observed between a large microbial cluster, including *UBA3282*, *Kineothrix*, *Muribaculum*, and *Prevotella*, and body weight, while another distinct group, including *Ruminococcus C 59129*, *Duncaniella*, and *Alistipes A 871400*, was strongly negatively correlated with TC, TG, and LDL levels (Figure 6C). Lastly, the combined intervention of PSRW and simvastatin induced the most distinct correlational patterns. A large bacterial cluster, including *Lactobacillus*, *Bacteroides H 857956*, and *Mailhella*, showed a strong positive correlation with glucose, BUN, and AST levels. Conversely, a separate group, including *Lepagella* and *Dysosmobacter*, was negatively correlated with AST. A negative correlation was also observed between *Allobaculum* and creatinine, and between *UBA3263* and TC levels (Figure 6D).

### 3.5. Effect of PSRW on the Predicted Functional Analysis of Gut Microbiota

**Figure 7:**
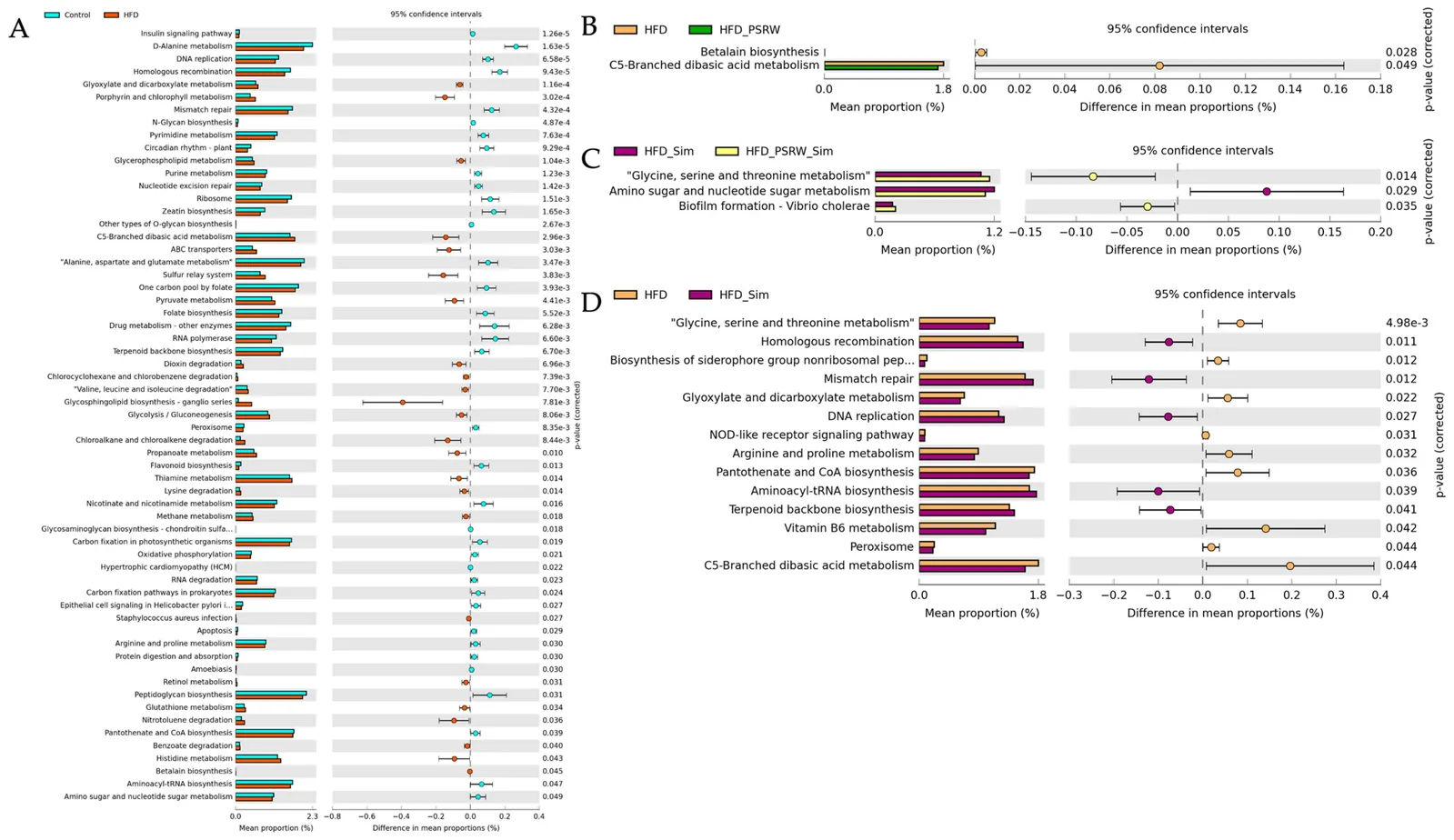
Predicted functional pathways of bacterial communities among experimental groups. (**A**) control vs. HFD, (**B**) HFD vs. HFD + PSRW, (**C**) HFD + Sim vs. HFD + PSRW + Sim, and (**D**) HFD vs. HFD + Sim.

To evaluate the functional potential of the gut microbiota, PICRUSt2 analysis was performed using the KEGG database to predict KEGG orthologs (KOs) and associated metabolic pathways. A total of 61 microbial metabolic pathways differed significantly between the control and HFD groups. For instance, glycosphingolipid biosynthesis was enriched in the HFD group, whereas D-alanine metabolism was significantly enriched in the control group (Figure 7A). When comparing the HFD and HFD + PSRW groups, dibasic acid metabolism was overrepresented in the HFD group, suggesting that PSRW supplementation suppressed this pathway (Figure 7B). In comparisons between the HFD and HFD + Sim groups, five pathways were significantly enriched in the HFD + Sim group, including mismatch repair, aminoacyl-tRNA biosynthesis, homologous recombination, DNA replication, and terpenoid backbone biosynthesis; conversely, the HFD group showed greater enrichment of dibasic acid metabolism, vitamin B6 metabolism, pantothenate and CoA biosynthesis, and amino acid metabolism (Figure 7D). Additionally, glycine, serine, and threonine metabolism was markedly enriched in the HFD + PSRW + Sim group, which is consistent with a possible complementary effect of PSRW and simvastatin on amino acid metabolism (Figure 7C).

## 4. Discussion

This study investigated the potential effect of *Piper sarmentosum* Roxb. aqueous extract on gut microbiota composition in a high-fat diet-induced obese rat model. The HFD successfully induced obesity-associated metabolic dysfunction, as indicated by significant weight gain, hyperlipidemia, and elevated liver enzymes (AST and ALT), indicative of hepatic stress and metabolic dysregulation [4,38]. Although PSRW treatment did not induce weight loss, it demonstrated potent metabolic benefits, significantly lowering serum TC, AST, and ALT relative to HFD, and TG relative to control. This suggests that the therapeutic action of PSRW may improve lipid metabolism and liver function directly, rather than through pathways controlling body mass or appetite. These beneficial effects may be attributed to bioactive compounds present in PSRW, particularly flavonoids, phenolic compounds, and tannins, which are known for antioxidant, antibacterial, anti-cancer, hypoglycemic, antihypertensive, and anti-inflammatory activities [39,40,41]. HPLC characterization of the same aqueous extract in our recent companion study confirmed that this metabolic phenotype rests on a defined phenolic profile, dominated by quercetin/trans-cinnamic acid, chlorogenic acid, caffeine, and gallic acid [33]. Quercetin and chlorogenic acid are well-recognized inhibitors of intestinal cholesterol micelle formation and bile-acid binding; gallic acid down-regulates PPARα-driven β-oxidation in NAFLD models; and epicatechin reduces oxidative stress in hepatocytes. Together, these constituents provide a plausible chemical rationale for the lipid- and liver-protective effects observed here [33].

A key finding of this study is that the therapeutic efficacy of PSRW in ameliorating dyslipidemia and hepatotoxicity was comparable to that of simvastatin, a potent HMG-CoA reductase inhibitor widely prescribed to lower endogenous cholesterol synthesis [42]. However, the PSRW extract exhibited an effect on TG metabolism, significantly lowering TG levels where simvastatin alone did not. Notably, the co-administration of PSRW and simvastatin exhibited an additive effect in reducing TC and hepatic enzyme levels. This interaction suggests that PSRW may complement and extend the efficacy of statin therapy in managing hyperlipidemia and potentially attenuate non-alcoholic fatty liver disease (NAFLD) [43,44,45]. This apparent complementarity may be explained by PSRW and simvastatin acting on largely non-overlapping nodes of lipid and microbial metabolism. Simvastatin primarily inhibits hepatic HMG-CoA reductase, blocking endogenous cholesterol synthesis. The known phenolic constituents of PSRW act extra-hepatically by (i) increasing intestinal cholesterol micelle size and decreasing micellar cholesterol solubility, (ii) binding bile acids (primarily taurocholic acid) and thereby reducing the reabsorbable bile-acid pool, (iii) down-regulating intestinal HMGCR while up-regulating cholesterol-efflux transporters ABCG5/ABCG8 and the transcription factor LXRα, and (iv) decreasing intestinal and hepatic [^3^H]-cholesterol accumulation to a degree comparable to ezetimibe, as we and others have shown [33]. This intestine-centric, microbiota-modulatory mode of action complements rather than duplicates the hepatic HMG-CoA blockade of simvastatin, providing a coherent mechanistic basis for the greater effect observed on TC and liver enzymes. The unique enrichment of glycine, serine, and threonine metabolism in the combination group reinforces this complementarity by supplying the carbon and methyl donors required for hepatic glutathione synthesis, one-carbon metabolism, and bile-acid conjugation, thereby reducing oxidative stress and supporting the disposal of cholesterol catabolites. Similar metabolic complementarity has been proposed for nutraceutical adjuncts in statin-intolerant patients and for rosuvastatin-related metabolic remodeling in obese individuals [9,10,43].

The disruption of gut microbial balance plays a major role in the development of obesity and metabolic disorders [12,13]. This imbalance substantially impacts host energy homeostasis by enhancing the metabolic harvest of dietary polysaccharides, regulating appetite, and controlling fat accumulation [41,46]. In the current study, the HFD group revealed a reduction in microbial richness, as indicated by a decreased Chao1 index, while Shannon evenness remained unaffected. Notably, no significant differences in overall alpha diversity were observed among the HFD-treated groups. In contrast, beta diversity showed distinct clustering between HFD and control groups, indicating substantial alterations in microbial community composition. Importantly, PSRW in HFD-fed rats partially reversed these compositional shifts, with microbial profiles clustering closely to those of the control, which may indicate that PSRW exerts its beneficial effects by remodeling the gut microbiome and fostering a more eubiotic state [45,47]. However, because these data are correlative, the direction of causality cannot be established: PSRW polyphenols may act directly on host lipid metabolism [48], with the microbial changes arising secondarily, or both may occur in parallel. Confirming a direct host effect would require germ-free or antibiotic-depletion models. However, simvastatin itself is not a purely lipid-lowering comparator. It also exerts pleiotropic anti-inflammatory actions and can modulate gut microbial composition [36,37]. These actions may therefore contribute to the effects observed in the combination group.

At the phylum level, we observed nuanced changes that challenge conventional biomarkers of dysbiosis. The ratio of *Bacillota* to *Bacteroidota* is often considered a key biomarker of susceptibility to obesity; however, our data did not show a significant difference in this ratio between groups, supporting a growing body of evidence that its utility as a universal marker is limited [49,50]. Instead, we observed a more pronounced effect on other phyla. The HFD group showed an increased relative abundance of *Desulfobacterota_G_459543* and *Pseudomonadota*, which have been positively associated with pro-inflammatory cytokines, including IL-6, IL-1β, and TNF-α, and with metabolic endotoxemia. Supplementation with PSRW reduced the prevalence of these taxa, suggesting a potential anti-inflammatory effect mediated through modulation of the gut microbiota [51,52]. We prioritized *Desulfobacterota* and *Pseudomonadota* over the conventional *Bacillota*/*Bacteroidota* ratio because, in similar HFD-induced dysbiosis models, these phyla are reproducibly enriched and have been mechanistically linked to obesogenic and inflammatory phenotypes: *Desulfobacterota* generate hydrogen sulfide that disrupts intestinal mucus integrity and promotes low-grade endotoxemia, whereas *Pseudomonadota* (formerly *Proteobacteria*) expansion is now considered a near-canonical signature of an unstable, inflammation-prone gut microbiota [51,52].

At the genus level, several bacterial taxa were associated with HFD-induced dysbiosis, including members of the *Lachnospiraceae* family such as *UBA3402* and *Schaedlerella*, as well as *Clostridium_AQ*, *Lawsonibacter*, *Monoglobus*, *Anaerotignum 189125*, and *Faecalibacterium*. Notably, the relative abundance of these genera was reduced under PSRW administration. These genera have been documented in HFD- and inflammation-context studies as opportunistic taxa linked to the development of obesity (e.g., expansion of *Schaedlerella* and *Lawsonibacter* in HFD-induced colitis and adiposity models; depletion of butyrate-producing *Faecalibacterium* in dysbiotic, metabolically unhealthy phenotypes), suggesting a potential protective mechanism of PSRW via suppression of obesogenic and pro-inflammatory microbes [53,54,55,56]. *Dwaynesavagella*, a member of the *Clostridiaceae* family, has been reported to confer health benefits, including activation of intestinal mucosal barrier integrity and homeostasis. In this study, PSRW supplementation significantly enriched the abundance of *Dwaynesavagella*, which may represent a beneficial microbe promoted by this intervention to counteract obesity development. Interestingly, both simvastatin and the combination therapy also modulated the abundance of *Faecalibacterium*, *Anaerotignum*, and *Bittarella*, which are known to improve glucose metabolism and exert anti-inflammatory effects, further highlighting the gut-centric action of these interventions [57,58,59].

Distinct clusters of microbes, associated with *Blautia A 141781* and *Faecousia*, showed a strong positive correlation with blood glucose, suggesting they may contribute to hyperglycemic conditions. Furthermore, *Mailhella* and *Paramuribaculum* were positively correlated with elevated TC and TG levels, directly linking them to dyslipidemia. *Kineothrix*, *Cryptobacteroides*, *Anaerotignum 189125*, *Ruminiclostridium E*, and *Romboutsia B* displayed a strong negative correlation with the liver enzyme AST, indicating that higher abundances of these microbes are associated with lower levels of liver damage and suggesting a protective role that is potentially insufficient in the dysbiotic HFD state. The intervention with PSRW dramatically reshaped these correlational patterns, indicating a profound functional shift in the gut environment. The most compelling evidence for the beneficial effect of PSRW is the emergence of new, protective associations: *Kineothrix* and *Muribaculum*, the latter previously linked to higher cholesterol in the HFD group, became significantly negatively correlated with AST and body weight [60,61,62,63]. This reversal is a key finding, suggesting that PSRW modulates the functional activity of specific taxa, transforming their association from detrimental to one that promotes lower liver stress and reduced body weight. Interestingly, the PSRW intervention did not simply reverse all HFD-induced correlations but established a new, distinct metabolic landscape, in which *Ruminococcus*, *Blautia*, *Phascolarctobacterium*, *Lawsonibacter*, *Treponema_D*, and *Cryptobacteroides* became strongly and negatively correlated with glucose and lipid profiles. This may reflect a shift in lipid transport and metabolism that contributes to the overall systemic reduction in serum TC and TG observed in the treated group [64,65,66,67].

To provide a mechanistic basis for these findings, a functional pathway analysis using PICRUSt2 was conducted. The HFD was associated with an enrichment of glycosphingolipid biosynthesis, a pathway strongly implicated in the pathogenesis of obesity and insulin resistance [68,69]. PSRW supplementation appeared to counteract HFD-induced metabolic shifts by suppressing pathways such as dibasic acid metabolism, whereas simvastatin treatment enriched pathways related to DNA repair and replication. Most notably, the combination of PSRW and simvastatin uniquely enhanced glycine, serine, and threonine metabolism, which may indicate a complementary effect whereby the combination therapy modulates key amino acid pathways essential for systemic metabolic health, potentially through roles in one-carbon metabolism and antioxidant defense. These distinct functional predictions provide a powerful mechanistic explanation for the therapeutic benefits observed [70,71,72,73]. Collectively, this study suggests that PSRW is a promising natural-product intervention for managing metabolic dysregulation; by improving key markers of glucose and lipid metabolism, PSRW could prevent and/or delay the onset of complex metabolic diseases, including type 2 diabetes and NAFLD.

### 4.1. Translational Considerations and Positioning as an Adjunct Strategy

The translation of these findings to human metabolic disorders requires careful consideration of three factors. (i) Dose translation: The PSRW dose used was 1000 µg/kg body weight/day by oral gavage, which relates to a human-equivalent dose of approximately 0.16 mg/kg/day (≈9.7 mg for a 60 kg adult) by body-surface-area conversion, based on a Km ratio for rats of 0.162 [74]. Although *P. sarmentosum* preparations are traditionally consumed as an edible plant, reliable per-serving intake data are not available, so direct dietary equivalence cannot be established, and dose-finding pharmacokinetic studies in humans are therefore required. (ii) Bioavailability: The active polyphenols in aqueous PSRW (quercetin glycosides, chlorogenic acid, epicatechin) show low intestinal absorption and extensive first-pass glucuronidation/sulfation. This profile is consistent with our hypothesis that much of the benefit of PSRW is mediated locally in the gut lumen (via direct microbiota modulation, bile-acid binding, and cholesterol micelle disruption) rather than through high systemic exposure to parent compounds. (iii) Inter-species differences in gut microbiota composition: rats and humans share many functional capacities but differ at the genus and species levels (e.g., the *Muribaculaceae* family is rodent-dominant), so the reversal of specific taxa here should be interpreted as evidence for functional reprogramming of the microbiome rather than direct one-to-one transferability to human taxa. Within these caveats, the data presented here, together with the in vitro and in vivo cholesterol-absorption evidence reported in our companion study [33], support positioning PSRW as a candidate adjunct to standard statin therapy for patients with hyperlipidemia and early-stage NAFLD, and as a preventive nutraceutical strategy in pre-obese, dysbiotic populations. The complementary mechanism (intestinal/microbial vs. hepatic) also raises the possibility of statin dose-sparing, which would be particularly relevant in statin-intolerant patients [9,10,43].

### 4.2. Limitations

Several limitations of this study should be acknowledged. (i) The modest group size (*n* = 5 per group), although justified a priori by power calculation for primary lipid endpoints and aligned with the 3Rs principle, limits the detection of small inter-group differences, particularly in compositional microbiome variables. (ii) Only male Wistar rats were studied; given the known sex differences in lipid metabolism, hepatic steatosis, and gut microbiota, the present findings cannot be generalized to female biology, and a sex-stratified follow-up is planned. (iii) A single PSRW concentration (1000 µg/kg bw/day, oral gavage) was tested; a full dose–response and pharmacokinetic characterization remains outstanding. (iv) Simvastatin at 40 mg/kg is not a purely lipid-lowering comparator: at this dose it also exerts anti-inflammatory activity and modulates gut microbial composition [36,37], so the effects observed in the combination group may partly reflect these pleiotropic actions of simvastatin rather than an interaction between PSRW and lipid lowering alone. (v) Obesity-specific endocrine biomarkers (leptin, adiponectin), insulin-sensitivity tests (GTT, ITT), and quantitative feeding-behavior/energy-expenditure measurements were not performed; their absence limits causal inference between microbiome reprogramming and adiposity. (vi) Food and water intake were measured at the cage level, as some rats were pair-housed; individual differences arising from housing conditions could have influenced blood parameters or gut microbiota composition and cannot be fully excluded. (vii) Although the marked normalization of serum AST and ALT, together with the cholesterol-lowering effect shown for PSRW in hepatic tissue by our companion study [33], strongly supports a hepatoprotective effect, formal histopathological grading of hepatic steatosis, inflammation, and fibrosis (e.g., NAS score), as well as of adipose-tissue morphology were not included and will be added in the planned confirmatory study, together with hepatic and adipose tissue antioxidant parameters (MDA, GSH, SOD, and CAT). (viii) Conventional status rather than SPF rats were used, and fecal sampling was performed only at the study endpoint; we therefore have no baseline (week 0) or post-induction (week 12) microbiome data and cannot verify that the groups were microbiologically comparable before treatment. (ix) The PICRUSt2-based functional predictions are inferred from 16S rRNA data and would benefit from confirmation by shotgun metagenomics and untargeted metabolomics in future work. (x) Correlations between specific gut microbes and metabolic markers are associative; causality will require fecal microbiota transplantation and germ-free re-colonization experiments.

## 5. Conclusions

In this HFD-induced obese rat model, the aqueous extract of *Piper sarmentosum* Roxb. (PSRW), whose phenolic composition was independently characterized by HPLC in our companion study [33], exerted a defined and reproducible metabolic phenotype: significant reduction in serum TC, AST, and ALT with efficacy comparable to simvastatin, and significantly lower TG than control, though not significantly different from HFD. Co-administration of PSRW and simvastatin produced a greater reduction in TC and liver enzymes than either agent alone, which may reflect an additive or synergistic interaction. At the gut microbiota level, PSRW partially reversed HFD-induced dysbiosis by suppressing pro-inflammatory phyla (*Desulfobacterota*, *Pseudomonadota*) and obesogenic *Lachnospiraceae* genera, while enriching beneficial *Dwaynesavagella* and functionally reprogrammed taxa such as *Kineothrix* and *Muribaculum* from metabolically detrimental to protective associations. PICRUSt2 analysis identified glycine–serine–threonine metabolism as a uniquely enriched pathway under combination therapy, providing a coherent one-carbon/antioxidant rationale for the enhanced effect of combination therapy with simvastatin. These findings position PSRW as a candidate intestine- and microbiota-associated phytotherapeutic that may complement the hepatic HMG-CoA inhibition of statins, with translational potential as an adjunct in dyslipidemia, early-stage NAFLD, and metabolic syndrome, particularly in statin-intolerant populations. Future work will (i) establish a full dose–response, (ii) include female animals and obesity-specific endocrine biomarkers (leptin, adiponectin), (iii) add hepatic and adipose histopathology with tissue antioxidant profiling, (iv) confirm causality through fecal microbiota transplantation, (v) validate the predicted functional pathways using shotgun metagenomics and untargeted metabolomics, and (vi) translate the most promising findings into a small-scale clinical study in patients with mild-to-moderate hyperlipidemia.

## Figures and Tables

**Table 1:** Effects of PSRW on body weight, lipid profile, liver enzymes, and kidney-function parameters.

Plasma Metabolic Parameter	Control	PSRW	HFD	HFD + PSRW	HFD + Sim	HFD + Sim + PSRW
Body weight (g)	750.00 ± 60.93	814.00 ± 106.74	1039.00 ± 72.49 ^a^	993.00 ± 73.11	979.00 ± 87.92	1005.00 ± 64.55
Glucose (mg/dL)	200.42 ± 39.18	198.97 ± 54.18	190.14 ± 34.13	198.49 ± 39.85	184.00 ± 36.28	204.19 ± 38.33
TC (mg/dL)	74.00 ± 12.85	81.29 ± 14.71	191.29 ± 68.13 ^a^	114.57 ± 26.39 ^b^	108.14 ± 18.47 ^b^	98.86 ± 6.25 ^b^
TG (mg/dL)	109.43 ± 32.86	96.00 ± 30.33	103.86 ± 26.67	72.29 ± 25.10 ^a^	80.29 ± 8.69	83.50 ± 23.45
HDL (mg/dL)	25.50 ± 6.47	18.17 ± 5.67	23.33 ± 9.53	24.00 ± 6.36	21.00 ± 1.77	22.25 ± 4.54
LDL (mg/dL)	74.61 ± 16.47	81.14 ± 18.81	130.43 ± 30.83 ^a^	104.49 ± 17.77	103.24 ± 14.19	87.18 ± 19.10
AST (U/L)	76.42 ± 9.68	80.58 ± 18.77	243.17 ± 89.95 ^a^	90.67 ± 8.81 ^b^	98.92 ± 13.15 ^b^	89.27 ± 7.78 ^b^
ALT (U/L)	93.75 ± 13.77	90.83 ± 9.03	250.42 ± 29.94 ^a^	107.92 ± 24.28 ^b^	120.83 ± 17.01 ^b^	96.77 ± 9.97 ^b^
BUN (mg/dL)	15.07 ± 4.82	18.62 ± 10.96	15.16 ± 2.67	13.71 ± 2.90	15.62 ± 2.87	13.92 ± 3.49
Creatinine (mg/dL)	0.66 ± 0.28	0.65 ± 0.26	0.81 ± 0.59	0.65 ± 0.31	0.74 ± 0.33	0.53 ± 0.45

Values are presented as mean ± SD; each group included *n* = 5 rats. PSRW: *P. sarmentosum* Roxb. extract; HFD: high-fat diet; Sim: simvastatin; TC: total cholesterol; TG: triglyceride; HDL: high-density lipoprotein; LDL: low-density lipoprotein; AST: aspartate aminotransferase; ALT: alanine aminotransferase; BUN: blood urea nitrogen. ^a^ significant difference from the control group; ^b^ significant difference from HFD. The mean difference is significant at the 0.05 level based on ANOVA with Bonferroni correction.

## Data Availability

The data supporting the findings of this study are openly available in the Zenodo repository at https://doi.org/10.5281/zenodo.16778463.

## Abbreviations

The following abbreviations are used in this manuscript:

ABCG5/ABCG8	ATP-binding cassette subfamily G member 5/8
ALT	Alanine aminotransferase
AST	Aspartate aminotransferase
BUN	Blood urea nitrogen
CAT	Catalase
GSH	Glutathione
GTT	Glucose tolerance test
HDL	High-density lipoprotein
HFD	High-fat diet
HMG-CoA	3-hydroxy-3-methylglutaryl coenzyme A
HPLC	High-performance liquid chromatography
ITT	Insulin tolerance test
KEGG	Kyoto Encyclopedia of Genes and Genomes
LDL	Low-density lipoprotein
MDA	Malondialdehyde
NAFLD	Non-alcoholic fatty liver disease
NAS	NAFLD activity score
PCoA	Principal coordinate analysis
PERMANOVA	Permutational multivariate analysis of variance
PICRUSt2	Phylogenetic Investigation of Communities by Reconstruction of Unobserved States 2
PSRW	*Piper sarmentosum* Roxb. aqueous extract
SCFA	Short-chain fatty acid
SD	Standard deviation
Sim	Simvastatin
SOD	Superoxide dismutase
TC	Total cholesterol
TG	Triglyceride
